# Alzheimer’s disease marker phospho-tau181 is not elevated in the first year after moderate-to-severe TBI

**DOI:** 10.1136/jnnp-2023-331854

**Published:** 2023-10-13

**Authors:** Neil Graham, Karl Zimmerman, Amanda J Heslegrave, Ashvini Keshavan, Federico Moro, Samia Abed-Maillard, Adriano Bernini, Vincent Dunet, Elena Garbero, Giovanni Nattino, Arturo Chieregato, Enrico Fainardi, Camelia Baciu, Primoz Gradisek, Sandra Magnoni, Mauro Oddo, Guido Bertolini, Jonathan M Schott, Henrik Zetterberg, David Sharp

**Affiliations:** 1 Brain Sciences, Imperial College London, London, UK; 2 UK Dementia Research Institute Centre for Care Research and Technology, Imperial College London, London, UK; 3 UK Dementia Research Institute, University College London, London, UK; 4 Dementia Research Centre, UCL Queen Square Institute of Neurology, London, UK; 5 Laboratory of Acute Brain Injury and Neuroprotection, Department of Acute Brain and Cardiovascular Injury, Istituto di Ricerche Farmacologiche Mario Negri IRCCS, Milan, Italy; 6 Dipartimento di Anestesia e Rianimazione, Fondazione IRCCS Ca' Granda Ospedale Maggiore Policlinico, Milan, Italy; 7 Neuroscience Critical Care Research Group, Department of Intensive Care Medicine, CHUV Lausanne University Hospital and University of Lausanne, Lausanne, Switzerland; 8 Department of Clinical Neurosciences, CHUV Lausanne University Hospital and University of Lausanne, Lausanne, Switzerland; 9 Department of Medical Radiology, CHUV Lausanne University Hospital and University of Lausanne, Lausanne, Switzerland; 10 Laboratory of Clinical Epidemiology, Istituto di Ricerche Farmacologiche Mario Negri IRCCS, Ranica, Italy; 11 Terapia Intensiva ad indirizzo Neurologico & Neurochirurgico, ASST Grande Ospedale Metropolitano Niguarda, Milan, Italy; 12 Department of Experimental and Clinical Sciences, Careggi University Hospital and University of Firenze, Florence, Italy; 13 Clinical Department of Anaesthesiology and Intensive Therapy, University Medical Center, Ljubljana, Slovenia; 14 Department of Anesthesia and Intensive Care, Santa Chiara Hospital, Trento, Italy; 15 Directorate for Innovation and Clinical Research, CHUV Lausanne University Hospital, Lausanne, Switzerland; 16 Dementia Research Centre and Department of Neurodegenerative Disease, UCL Queen Square Institute of Neurology, London, UK; 17 Department of Psychiatry and Neurochemistry, The Sahlgrenska Academy at the University of Gothenburg, Mölndal, Sweden

**Keywords:** TRAUMATIC BRAIN INJURY, DEMENTIA

## Abstract

**Background:**

Traumatic brain injury (TBI) is associated with the tauopathies Alzheimer’s disease and chronic traumatic encephalopathy. Advanced immunoassays show significant elevations in plasma total tau (t-tau) early post-TBI, but concentrations subsequently normalise rapidly. Tau phosphorylated at serine-181 (p-tau181) is a well-validated Alzheimer’s disease marker that could potentially seed progressive neurodegeneration. We tested whether post-traumatic p-tau181 concentrations are elevated and relate to progressive brain atrophy.

**Methods:**

Plasma p-tau181 and other post-traumatic biomarkers, including total-tau (t-tau), neurofilament light (NfL), ubiquitin carboxy-terminal hydrolase L1 (UCH-L1) and glial fibrillary acidic protein (GFAP), were assessed after moderate-to-severe TBI in the BIO-AX-TBI cohort (first sample mean 2.7 days, second sample within 10 days, then 6 weeks, 6 months and 12 months, n=42). Brain atrophy rates were assessed in aligned serial MRI (n=40). Concentrations were compared patients with and without Alzheimer’s disease, with healthy controls.

**Results:**

Plasma p-tau181 concentrations were significantly raised in patients with Alzheimer’s disease but not after TBI, where concentrations were non-elevated, and remained stable over one year. P-tau181 after TBI was not predictive of brain atrophy rates in either grey or white matter. In contrast, substantial trauma-associated elevations in t-tau, NfL, GFAP and UCH-L1 were seen, with concentrations of NfL and t-tau predictive of brain atrophy rates.

**Conclusions:**

Plasma p-tau181 is not significantly elevated during the first year after moderate-to-severe TBI and levels do not relate to neuroimaging measures of neurodegeneration.

## Introduction

Traumatic brain injury (TBI) is common occurrence and an environmental risk factor for dementia. A range of pathologies are described postinjury including the tauopathies Alzheimer’s disease (AD) and chronic traumatic encephalopathy (CTE),^1^ which form part of the broader complex constellation of postinjury pathologies and has been termed traumatic brain injury-related neurodegeneration (TReND) (online supplemental ref).

10.1136/jnnp-2023-331854.supp1 Supplementary data

A key question is how acute traumatic elevations of τ relate to progressive neurodegeneration. This is potentially mechanistically important as abnormal τ may cause neurodegeneration through prion-like proteopathic seeding. Total τ (t-tau) is increased more than one hundred-fold after moderate-to-severe TBI in brain extracellular fluid, and plasma t-tau predicts neurodegeneration. However, in contrast to the axonal degeneration marker neurofilament light (NfL) and astroglial marker glial fibrillar acidic protein (GFAP), t-tau rapidly normalises.^2^ Advanced biomarker assays now allow investigation of links between TBI and dementia. In AD, τ phosphorylated at threonine 181 (p-tau_181_) correlates with neuropathology,^3^ and is being incorporated clinically. P-tau_181_ has not been investigated after moderate-to-severe TBI.

We assessed plasma P-tau_181_ in a subset of the BIO-AX-TBI cohort of moderate-to-severe TBI (defined using the Mayo classification, see online supplemental file 1), healthy controls and AD patients, comparing blood biomarker concentrations to MRI measures of neurodegeneration in the TBI group. We have previously described the cohort and characterised trends of NfL, t-tau, neuronal marker ubiquitin C-terminal hydrolase L1 (UCH-L1) and GFAP. Hence, we were able to directly compare these markers with P-tau_181_ .^2^ We hypothesised that: (1) p-tau_181_ would increase early post-TBI, (2) remain elevated at 1 year and (3) predict brain atrophy.

## Methods

See online supplemental file 1.

## Results

P-tau_181_ was quantified over 1 year in 42 patients after moderate-to-severe TBI, aged 48.7 years (mean, SD 15.6) with 76.2% male (online supplemental table 1). The lowest Glasgow Coma Scale was 3–8 in 26.8%, 9–13 in 39.0% and 14–15 in 34.1% (unknown in n=1). Diffuse injury was present on CT in 4.8%, with contusions/intraparenchymal haemorrhage in 47.6%, subdural haematoma in 57.1%, and extradural haematoma in 14.3%. This group had less severe injuries that the broader BIO-AX-TBI cohort previously reported, indicated by lower peak 10-day injury biomarker concentrations (all p<0.05, except NfL).^2^ A group of male healthy controls underwent aligned p-tau_181_ assessment (mean 45.9 years, SD 3.2). Thirty-one patients with AD were also assessed (63.7 years, SD 6.5, 48.4% male) and 14 non-AD controls (mean 60.6 years, SD 6.5, 71.4% male). Age and sex were included as confounders.

**Figure 1:**
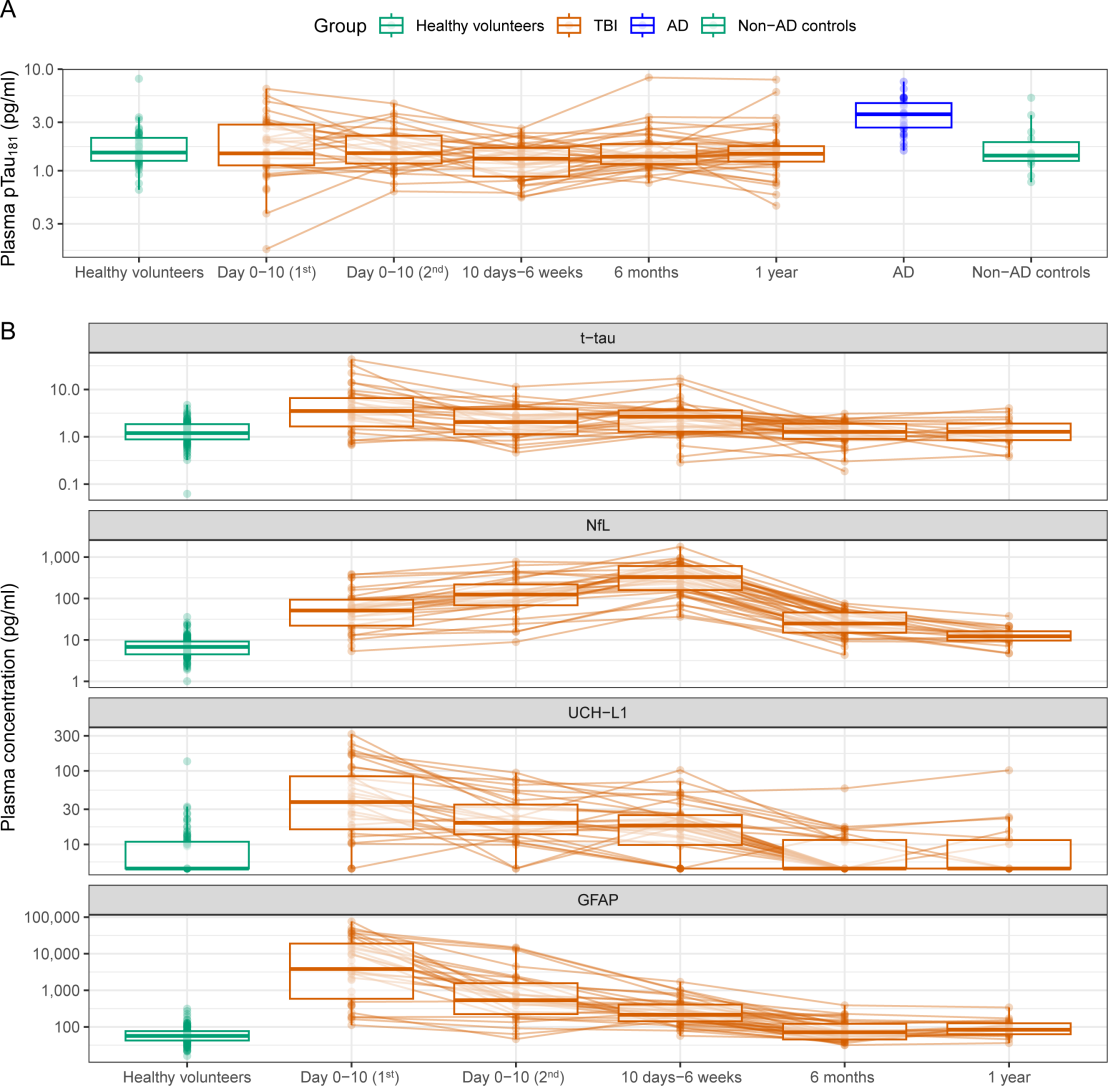
Longitudinal fluid biomarker trajectories after moderate-to-severe TBI, healthy volunteers and Alzheimer’s disease. Fluid biomarkers in healthy volunteers, longitudinally in patients following TBI, people with AD and non-AD healthy controls (non-AD controls). Boxplots show median and quartiles (hinges), with whiskers extending upto 1.5 times the IQR. Individual data points are shown and connected by lines indicating within-subject trajectories. (A) shows p-tau_181_; (B) shows total τ (t-tau), neurofilament light (NfL), ubiquitin c-terminal hydrolase L1 (UCH-L1) and glial fibrillar acidic protein (GFAP). AD, Alzheimer’s disease; TBI, traumatic brain injury.

Median plasma p-tau_181_ at 2.7 days (SD 2.8) post-TBI was 1.5 pg/mL (IQR 1.7) and did not differ significantly from healthy volunteers (median 1.5 pg/mL, IQR 1.9) (see figure 1; online supplemental table 2). P-tau_181_ concentrations were stable in the year after injury: day 6.3 (SD 2.4) 1.5 pg/mL (IQR 1.0); day 30.0 (SD 12.1) 1.3 pg/mL (IQR 0.8); 6 months 1.4 pg/mL (IQR 0.7) and 1 year 1.5 pg/mL (IQR 0.5). There was no significant longitudinal change. In contrast, reductions were seen in t-tau, GFAP, NfL and UCH-L1 as previously reported (all p<0.001).^2^

There was no significant correlation of p-tau_181_ measured subacutely and grey matter atrophy measured at 6 months, nor between concentrations within 6 months and white matter atrophy at 6–12 months. As previously shown in the wider cohort,^2^ hyperacute t-tau predicted grey matter atrophy (p=0.046; adjusted R^2^=0.12), and subacute plasma NfL predicted white matter atrophy (p=0.004, R^2^=0.20).^2^ P-tau_181_ was significantly raised in AD (2.0 times (1.5–2.7) higher, p<0.001) versus non-AD controls.

## Discussion

Plasma p-tau_181_ was not elevated after moderate-to-severe TBI and concentrations did not vary significantly over 1 year. This contrasts with our previous findings in other markers t-tau, NfL, GFAP and UCH-L1, which showed substantial elevations.^2^ Unlike plasma t-tau and NfL, we found no correlation of p-tau_181_ at any time point and brain atrophy, a measure of neurodegeneration.

P-tau_181_ is a neuropathologically validated in vivo marker of amyloid-induced τ phosphorylation in AD.^3^ Given the presence of amyloid and τ pathologies post-TBI, there is interest in whether TBI may trigger neurodegeneration through mechanisms similar to AD, and whether this is reflected in AD-specific blood biomarkers. AD patients showed substantial p-tau_181_ elevations, demonstrating the assay’s sensitivity, but this was not seen post-TBI. The lack of p-tau_181_ elevation at 1-year contrasts with neurodegeneration marker NfL and astroglial marker GFAP, both of which remained chronically elevated, as previously reported.^2^ It is possible that phosphorylated τ accumulates as a late consequence of TBI and we would not have identified this in our 1-year follow-up period. Post-traumatic neurodegeneration is likely to be dynamic over time and a specific temporal pattern of plasma p-tau isoform changes, as seen in AD where plasma p-tau_231_ precedes p-tau_181_ positivity, with both markers well correlated with τ PET.^4^ Very long-term follow-up incorporating comprehensive longitudinal fluid biomarker assessment, brain volumetry and molecular imaging would likely be highly informative.

It is possible that other blood markers may be more specific to post-traumatic neurodegeneration. For example, postmortem work suggests that p-tau_202_ may have greatest specificity for CTE. This has yet to be assessed clinically as there is not currently a reliable assay to do so at scale. In addition, brain derived τ shows promise as a marker, correlating more closely with CSF τ and neurofibrillary tangle burden in AD better than t-tau (online supplemental ref).

There are several potential limitations. Relatively few patients were sampled <24 hours after injury, hence we may have missed an early peak in p-tau_181_ as previously seen using a different assay-type quantifying p-tau_231_.^5^ Second, the use of p-tau_181_ controls analysed separately from TBI patients may introduce bias: however, we feel this is unlikely due to good assay performance, large numbers, and lack of longitudinal injury-associated change. Last, TBI and young controls were not well sex-matched, though this was included in statistical models.

In conclusion, plasma p-tau_181_ was not increased over 1 year after moderate-to-severe TBI and was not associated with neurodegeneration. P-tau_181_ dynamics were not only distinct from t-tau but differed from other biomarkers NfL, GFAP and UCH-L1. This suggests that p-tau_181_ does not contribute to progressive neurodegeneration commonly seen after TBI, at least in the first year.
