# Supplementary material for: Alzheimer’s disease marker phospho-tau181 is not elevated in the first year after moderate-to-severe TBI
Source: J Neurol Neurosurg Psychiatry. 2023 Oct 13;95(4):356–9. doi: 10.1136/jnnp-2023-331854 (PMC10958285; doi:10.1136/jnnp-2023-331854)
Supplement: Supplementary data [file jnnp-2023-331854supp001.pdf]

# Alzheimer's disease marker phospho-tau181 is not elevated in the first year after moderate-severe TBI

Neil S.N. Graham[1,2,3], Karl A. Zimmerman[1,2,3], Amanda Heslegrave[4,5], Ashvini Keshavan[6], Federico Moro[7,8], Samia Abed Maillard[9], Adriano Bernini[10], Vincent Dunet[11], Elena Garbero[12], Giovanni Nattino[12], Arturo Chieregato[13], Enrico Fainardi[14], Camelia Baciu[13], Primoz Gradisek[15], Sandra Magnoni[16], Mauro Oddo[9,17], Guido Bertolini[12], Jonathan M Schott[4,5,6], Henrik Zetterberg[4,5,18,19,20,21], David J. Sharp*[1,2,3]

## SUPPLEMENTARY METHODS

The BIO-AX-TBI cohort [1] assessed axonal injury over one year in patients after moderate-severe TBI using advanced biomarkers, relating these to clinical outcomes. Patients presenting to major trauma centres at participating study sites were eligible for inclusion if they met the Mayo classification criteria for moderate-severe TBI.[2] These include: acute abnormality on CT (eg. subarachnoid haemorrhage, subdural haematoma, extradural haematoma, intraparenchymal bleed/contusion), injury penetrating the dura, post traumatic amnesia duration > 24 hours, loss of consciousness > 30 minutes, worst Glasgow Coma Scale < 13 or death due to TBI. The presence of any one of these features is sufficient for the patient to meet criteria for moderate-severe injury. Blood was sampled twice within ten days post-injury (first sample mean 2.7 days post-TBI [standard deviation 2.8], second sample 6.3 [2.4] days), between ten days and six weeks (26.0 [2.4] days since injury), at six months (189.0 [16.0] days) and twelve months (371.0 [55.2] days). Plasma was sampled using standard procedures and frozen at -80C. P-$tau_{181}$ was quantified using a single-plex P-tau181 V2.0-Advantage kit on a Simoa HD-X (Quanterix, Billerica, MA). UCH-L1, GFAP, total tau and NfL concentrations were previously measured using multiplexed kits (Neurology 4-Plex B, Quanterix, Billerica, MA).[3] A 1:4 dilution was used on-board the instrument. Samples were run in duplicate and average values reported. The intraplate coefficient of variation (CV) for p-$tau_{181}$ was 8.4% with an interplate CV of 5.9%.

Due to limitations in the $Ptau_{181}$ assay availability and the large number of longitudinal samples in BIO-AX-TBI cohort, we elected analyse a subset of the group where there were samples spanning an entire year, including: an acute blood sample within days 1-10, as well as a chronic sample at six and twelve months post-injury. This equated to n=42 individuals.



Graham N, et al. J Neurol Neurosurg Psychiatry 2024; 95:356–359. doi: 10.1136/jnnp-2023-331854

Healthy control data, comprising p-tau$_{181}$ concentrations from the ADVANCE cohort (50 male service personnel with no history of major trauma) were used for comparison.[4, 5] The intraplate CV was 8.3% and interplate CV was 8.6%. Data from a separate, pre-existing group of age-matched healthy controls in BIO-AX-TBI provided norms for previously tested biomarkers UCH-L1, GFAP, t-tau, NfL, using Neurology 4-plex B kits on a Simoa HD-1.

People with Alzheimer's disease (AD) and non-AD controls were assessed at UCL (Study 12/3044). Patients had a clinical diagnosis of Alzheimer's type mild cognitive impairment or dementia with CSF showing amyloid pathology (defined by amyloid beta 42:40 ratio $\leq$ 0.065). Age-matched controls had subjective cognitive impairment or primary mood disorder and all were amyloid-negative. Analysis was performed with a single-plex P-tau181 kit (intraplate CV 1.16%).

The Marshall classification defined acute imaging pathologies. MRI was acquired in 40 of the TBI patients. Pairwise volumetric T1-weighted MRI was analysed as previously [3] using SPM12 (UCL), producing grey/white matter atrophy rates spanning subacute (10 day-6 weeks to 6 months) and early-chronic (6 months to 12 months) periods.

Statistical analyses were conducted using R (v4.1.1). Group differences were assessed using linear regression; longitudinal biomarker assessment within TBI patients was performed using linear mixed effects modelling with subject as a random effect. Due to their non-normal distribution log-transformed biomarker concentrations were used; age and sex were included as covariates. The relevant research ethics committee approvals were granted for the investigation (UK REC ref: 17/LO/2066, Camberwell and St Giles Ethics Committee).

# SUPPLEMENTARY RESULTS

## Supplementary Table 1. Demographics and Plasma Biomarker Concentrations

Demographics and plasma concentrations of biomarkers reported for patients and controls. Values for patients after TBI are for the first acute testing timepoint only (ie. the first sample taken within ten days of injury). IQR: interquartile range; NfL: neurofilament light; UCH-L1: ubiquitin c-terminal hydrolase-L1; p-tau$_{181}$ :tau phosphorylated at serine 181, T-tau: total tau; GFAP: glial fibrillary acidic protein

| | | **Traumatic brain injury** | **Ptau181 healthy controls** | **Other biomarker healthy controls** | **Alzheimer's disease patients** | **Non-AD controls** |
|---|---|---|---|---|---|---|
| *Demographics* | n | 42 | 50 | 128 | 31 | 14 |
| | Age (mean (SD)) | 48.7 (15.7) | 45.9 (3.20) | 42.1 (16.8) | 63.7 (6.48) | 60.5 (6.50) |
| | Sex = Male (%) | 32 (76.2) | 50 (100.0) | 78 (60.9) | 15 (48.4) | 10 (71.4) |
| *Plasma biomarker concentration (pg/ml), (median [IQR])* | P-tau$_{181}$ | 1.48 [1.13, 2.85] | 1.51 [1.25, 2.11] | — | 3.60 [2.66, 4.64] | 1.41 [1.25, 1.93] |
| | T-tau | 3.50 [1.66, 6.57] | — | 1.20 [0.88, 1.85] | — | — |
| | NfL | 51.3 [22.1, 93.8] | — | 5.91 [4.12, 8.72] | — | — |
| | UCH-L1 | 37.6 [16.1, 84.5] | — | 4.69 [4.69, 10.88] | — | — |
| | GFAP | 3857 [589, 18942] | — | 57 [40, 78] | — | — |

**Supplementary Table 2. Longitudinal fluid biomarker concentrations following TBI**

IQR: interquartile range; NfL: neurofilament light; UCH-L1: ubiquitin c-terminal hydrolase-L1; p-tau$_{181}$: tau phosphorylated at serine 181; T-tau: total tau; GFAP: glial fibrillary acidic protein.

| | **Timepoint following moderate-severe TBI** | | | | |
|---|---|---|---|---|---|
| | Day 0-10, first sample | Day 0-10, second sample | Subacute, day 10-6 weeks | 6 month | 12 month |
| **P-tau$_{181}$** | | | | | |
| n | 37 | 30 | 42 | 42 | 42 |
| Median | 1.5 | 1.5 | 1.3 | 1.4 | 1.5 |
| IQR | 1.7 | 1 | 0.8 | 0.7 | 0.5 |
| **T-tau** | | | | | |
| n | 36 | 30 | 42 | 40 | 21 |
| Median | 3.5 | 2.1 | 2.7 | 1.3 | 1.3 |
| IQR | 4.9 | 2.8 | 2.3 | 1 | 1.1 |
| **NfL** | | | | | |
| n | 36 | 30 | 42 | 40 | 21 |
| Median | 51.3 | 124.7 | 328.4 | 24.9 | 12.2 |
| IQR | 71.7 | 152.6 | 445.6 | 30.9 | 6.5 |
| **UCH-L1** | | | | | |
| n | 36 | 30 | 42 | 37 | 18 |
| Median | 37.6 | 19.6 | 18 | 4.7 | 4.7 |
| IQR | 68.5 | 21.1 | 15.1 | 6.8 | 6.8 |
| **GFAP** | | | | | |
| n | 36 | 30 | 42 | 40 | 21 |
| Median | 3857 | 534.5 | 213.1 | 71.1 | 83.8 |
| IQR | 18352.7 | 1338.5 | 264.2 | 76 | 62.7 |

# REFERENCES

## Additional references (main manuscript)

Smith, D.H., et al., *COllaborative Neuropathology NEtwork Characterizing ouTcomes of TBI (CONNECT-TBI).* Acta Neuropathol Commun, 2021. **9**(1): p. 32

Gonzalez-Ortiz, F., et al., *Brain-derived tau: a novel blood-based biomarker for Alzheimer's disease-type neurodegeneration.* Brain, 2023. **146**(3): p. 1152-1165

## Supplementary references

1. Graham, N.S., et al., *Multicentre longitudinal study of fluid and neuroimaging BIOmarkers of AXonal injury after traumatic brain injury: the BIO-AX-TBI study protocol.* BMJ Open, 2020. **10**(11): p. e042093.
2. Malec, J.F., et al., *The mayo classification system for traumatic brain injury severity.* J Neurotrauma, 2007. **24**(9): p. 1417-24.
3. Graham, N.S.N., et al., *Axonal marker neurofilament light predicts long-term outcomes and progressive neurodegeneration after traumatic brain injury.* Science Translational Medicine, 2021. **13**(613): p. eabg9922.
4. Bennett, A.N., et al., *Study protocol for a prospective, longitudinal cohort study investigating the medical and psychosocial outcomes of UK combat casualties from the Afghanistan war: the ADVANCE Study.* BMJ Open, 2020. **10**(10): p. e037850.
5. Graham, N.S.N., et al., *ADVANCE-TBI study protocol: traumatic brain injury outcomes in UK military personnel serving in Afghanistan between 2003 and 2014 – a longitudinal cohort study.* BMJ Open, 2023. **13**(3): p. e069243.
6. Smith, D.H., et al., *COllaborative Neuropathology NEtwork Characterizing ouTcomes of TBI (CONNECT-TBI).* Acta Neuropathol Commun, 2021. **9**(1): p. 32.



Graham N, *et al. J Neurol Neurosurg Psychiatry* 2024; 95:356–359. doi: 10.1136/jnnp-2023-331854
